# Biosurfactant Mediated Biosynthesis of Selected Metallic Nanoparticles 

**DOI:** 10.3390/ijms150813720

**Published:** 2014-08-08

**Authors:** Grażyna A. Płaza, Joanna Chojniak, Ibrahim M. Banat

**Affiliations:** 1 Department of Environmental Microbiology, Institute for Ecology of Industrial Areas, 6 Kossutha Str., 40-844 Katowice, Poland; E-Mail: chojniak@ietu.katowice.pl; 2 School of Biomedical Sciences, University of Ulster, Coleraine BT52 1SA, N. Ireland, UK; E-Mail: IM.Banat@ulster.ac.uk

**Keywords:** nanobiotechnology, biosynthesis, metals nanoparticles (MeNPs), biosurfactants

## Abstract

Developing a reliable experimental protocol for the synthesis of nanomaterials is one of the challenging topics in current nanotechnology particularly in the context of the recent drive to promote green technologies in their synthesis. The increasing need to develop clean, nontoxic and environmentally safe production processes for nanoparticles to reduce environmental impact, minimize waste and increase energy efficiency has become essential in this field. Consequently, recent studies on the use of microorganisms in the synthesis of selected nanoparticles are gaining increased interest as they represent an exciting area of research with considerable development potential. Microorganisms are known to be capable of synthesizing inorganic molecules that are deposited either intra- or extracellularly. This review presents a brief overview of current research on the use of biosurfactants in the biosynthesis of selected metallic nanoparticles and their potential importance.

## 1. Introduction

The prefix “nano” is derived from Greek word “nanos” meaning “dwarf” that refers to things of one-billionth (10^−9^ m) in size. Nanostructures with at least one dimension between 1 and 100 nm have been attracting interest due to unique properties and wide applications in various fields of our daily activities. The provision of simple methods for the preparation of nano-sized metal particles, therefore, has attracted significant attention because of their numerous potential applications. Nanomaterials provide solutions to technological and environmental challenges in various fields such as energy, medicine, electronics, cosmetics, coatings, packaging and biotechnology. Research and development in nanotechnology is different to other fields of science. The characteristics of nanoparticles (NPs) are closely dependent upon their shape, overall size, size range distribution and composition [1].

Nanoparticles are usually synthesized using divers physical and chemical complex processes that use high temperature, pressure and energy and many biologically toxic compounds generating and introducing environmental pollutants [2,3]. The most popular method is the chemical reduction method, in which the metals salts are converted to metal atoms by using reducing agents such as citrate, hydrides, ethylene glycol and hydrazine, all of which pose environmental and health risks. Consequently, during the last decade, the biosynthesis of metal nanoparticles (MeNPs) has emerged and is undergoing development as an alternative environmentally benign procedure. The biological methods of nanoparticles synthesis belong to new green generation processes, which are eco-friendly and are designed as credible alternatives to chemical and physical methods often called “green-synthesis” or “green chemistry” procedures. Traditionally nanoparticles are commonly synthesized by two strategies: top-down and bottom-up approach [4]. In the top-down approach, the bulk materials are gradually broken down to nanosized materials whereas in bottom-up approach, atoms or molecules are assembled to molecular structures in the nano-meter range. The bottom-up approach is commonly used for chemical and biological synthesis of nanoparticles. 

Nanobiotechnology (bionanotechnology, nanobiology) areas of interest have emerged recently as a result of an active integration between microbial biotechnology and nanotechnology. Microbial biotechnology has traditionally used microorganisms and their products in various applications such as medicine, industry, food production, agriculture and remediation technologies. Heavy toxic metals are usually reduced to metal ions to precipitate them or to detoxify the environment they are present in. It has been reported that nanoparticles can be synthesized by biological sources such as plant extracts, fungi, algae, cyanobacteria, bacteria, yeasts, actinomycetes and standalone biomolecules [5,6,7,8,9,10,11,12]. Thakkar *et al.* [13] and Narayanan and Sakthivel [4] have provided the status of various biological syntheses of different metallic and bimetallic nanoparticles while most recently Quester *et al.* [14] summarize some of the most significant results using organisms to produce metallic NPs as well as the microscopic analyses to characterize the nanostructured material obtained, providing valuable materials for future research.

Ongoing research in this area is focused on finding biological protocols to resolve the polydispersity and gain control over the size and shape of nanostructures. The use of biological systems for the synthesis of NPs offers several advantages since the methods are easier to carry out and more economical, safer and environmentally benign compared to traditional production processes [13]. Many biologically synthesized nanoparticles have been reported as multi-functional particles with diverse biomedical applications (nanomedicine) [15]. 

The purpose of this review is to provide a comprehensive overview of advances in the applications of biosurfactants in biosynthesis of metallic nanoparticles.

## 2. Microbial Biosynthesis of Nanoparticles

Microbial synthesis of nanoparticles is a green chemistry approach that interconnects nanotechnology and microbial biotechnology. Biosynthesis of gold, silver, gold-silver alloy, selenium, tellurium, platinum, palladium and uraninite nanoparticles has been reported in the literature. Some examples of microorganisms reported for the production of different types of nanoparticles are presented in Table 1. Such biosynthesis of nanoparticles is carried out by microorganisms which grabs target ions from solutions and then accumulate the reduced metal in its elemental form through enzymatic activities (as bio-reducing agent) generated by microbial cells metabolic activities. It is then categorized as intracellular or extracellular synthesis according to where nanoparticles formation took place [8].

**Table 1:** List of microorganisms known to produced different nanoparticles modified from [13,16].

Microorganisms	Type of Nanoparticles
*Bacteria*	
*Bacillus subtilis*	Ag
*Pseudomonas stutzeri*	Ag, Au
*Pseudomonas aeruginosa*	Au
*Shewanella algae*	Au
*Shewanella oneidensis*	Uranium (IV)
*Lactobacillus* strains	Au, Ag, Au-Ag alloy, TiO_2_
*Clostridium thermoaceticum*	CdS
*Klebsiella aerogenes*	CdS, Ag
*Escherichia coli*	CdS, Au
*Rhodopseudomonas capsulata*	Au
*Desulfobacteriaceae*	ZnS
*Rhodococcus* strains	Au
*Yeast*	
*Candida glabrata*	CdS
*Torulopsis* sp.	PbS
*Schizosaccharomyces pombe*	CdS
*S. cerevisiae*	Sb_2_O_3_, TiO_2_
*Fungi*	
*Verticillium* sp.	Ag, Au
*Fusarium oxysporum*	Ag, Au, Au-Ag alloy, CdS
*Colletotrichum* sp*.*	Au
*Aspergillus fumigatus*	Ag
*Trichoderma asperellum*	Ag
*Phaenerochaete chrysosporium*	Ag
*Schizosaccharomyces cerevisiae*	Au
*Yarrowia lipolytica*	Au
*Torulopsis* sp*.*	PbS
*Candida glubrata*	CdS
*Actinomycetes*	
*Rhodococcus* sp.	Au
*Thermomonospora* sp.	Au
*Algae*	
*Chlorella vulgaris*	Au
*Phaeodactylum tricornutum*	CdS
*Sargassum wightii*	Au

It is well known that many organisms can provide inorganic materials. For example, magnetotactic bacteria produce magnetite nanoparticles and diatoms synthesize siliceous materials [16]. Many organisms produce bio-minerals such as gypsum and calcium carbonate layers which consist of an inorganic component in a special organic matrix (polysaccharides, lipids, proteins). Recently, these organisms are also described as “eco-friendly nano factories” used to produce various nanostructures. 

**Figure 1:**
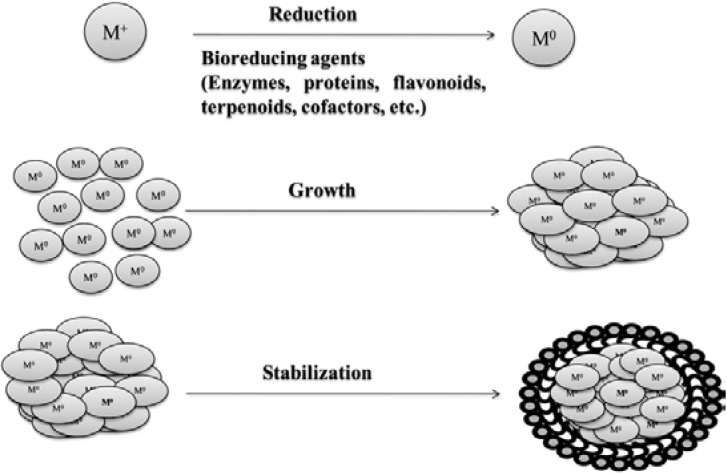
The formation of the metal nanoparticles (Me-NPs) during biosynthesis. Reprint from [12] with permission from Elsevier, copyright 2013. M^+^, metal ion; M^0^, reduced metal ion.

The general scheme of the formation of metallic nanoparticles through biosynthesis is shown in Figure 1. Mostly, two types of green synthesis of nanoparticles have so far been identified. The first option is the use of all microbial systems (all organisms) for the synthesis of nanoparticles and the second option is to use the metabolites synthesized by different organisms (microorganisms or plants). In the case of microorganisms, the nanoparticles are produced either intra- or extra-cellularly [17]. In case of the intracellular synthesis the nanoparticles are produced inside the bacterial cells by the reductive pathways of the cell wall and accumulated in the periplasmic space of the cell. The nanoparticles are produced extracellularly when the cell wall reductive enzymes or soluble secreted enzymes are extracted outside the cell and are involved in the reductive process of metal ions. The potential of biological materials for nanoparticles synthesis is quite wide due to the rich biodiversity of microbes. However, despite some related reports, many aspects of nanoparticles biosynthesis remain unclear especially with regards to why and how the size and shapes of the synthesized nanoparticles are influenced by the biological systems. 

**Figure 2:**
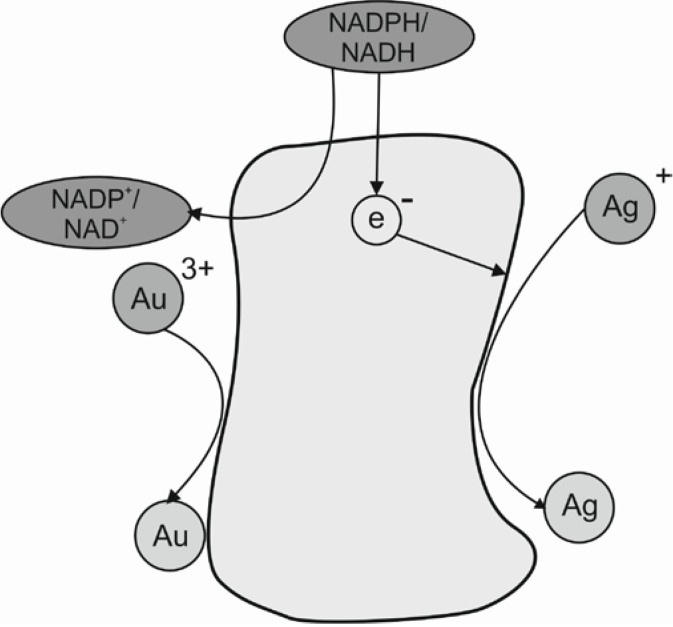
Hypothetical mechanism for silver and gold nanoparticles biosynthesis modified from [18] with permission from Elsevier, copyright 2007.

One of the enzymes involved in the biosynthesis of metal nanoparticles is the nitrate reductase which reduces the metal ions (Me^+1^) to the metallic form (Me^0^). This enzyme is a NADH^−^ and NADPH-dependent enzyme. He *et al.* [18] described the hypothetical mechanism for gold nanoparticles biosynthesis carried out by *Rhodopseudomonas capsulate*. These bacteria are known to secrete cofactor NADH- and NADH-dependent enzymes that can be responsible for the biological reduction of Au^3+^ to Au^0^ and the subsequent formation of gold nanoparticles. This reduction is initiated by electron transfer from the NADH by NADH-dependent reductase as electron carrier during which the gold ions gain electrons and are therefore reduced to Au^0^. A similar mechanism was also described by Sadowski [19] for silver nanoparticles synthesis by *Bacillus licheniformis*. The likely mechanism for the biosynthesis of silver and gold nanoparticles is presented in Figure 2. 

## 3. Biosurfactants—Types, Structures and Properties

Biosurfactants (biological surface active compounds or microbial surface active agents) are surface-active biomolecules produced by living cells, mainly by microorganisms [20]. They are amphiphilic biochemical compounds containing both hydrophobic and hydrophilic moieties that allow them to exist or partition at the interface between polar and nonpolar media [21]. They are produced by a variety of microorganisms, mainly bacteria, fungi and yeasts. Table 2 shows the major biosurfactant types and the microorganisms involved in their production. They are produced on microbial cell surfaces or are extracellularly secreted. Structurally, their hydrophilic moiety can be a carbohydrate, amino acid, cyclic peptide, phosphate, carboxylic acid or an alcohol and the hydrophobic moiety is mostly either, a long-chain fatty acid, hydroxyl fatty acid or α-alkyl β-hydroxy fatty acid [22]. 

**Table 2:** Various biosurfactants produced by microorganisms.

Biosurfactant Type	Microbial Species
*Glycolipids*	
Trehalose mycolates	*Rhodococcus erythropolis*, *Arthobacter paraffineu*, *Mycobacterium phlei*, *Nocardia erythropolis*
Trehalose esters	*Mycobacterium fortium*, *Micromonospora* sp., *M. smegmatis*, *M. paraffnicum*, *Rhodococcus erythropoli*, *Arthobacter* sp*.*, *Nocardia* sp.
Rhamnolipids	*Pseudomonas* spp., *Pseudomonas chlororaphis*, *Burkholderia* spp.
Sophorolipids	*Candida bombicola*/*apicola*, *Torulopsis petrophilum*, *Candida* sp., *Candida antartica*, *Candida botistae*, *Candida riodocensis*, *Candida stellata*, *Candida bogoriensis*
Flocculosin	*Pseudomonas flocculosa*
*Phospholipids and fatty acids*	
Phospholipids, Fatty acids	*Candida* sp., *Corynebacterium* sp., *Micrococcus* sp., *Acinetobacter* sp., *Thiobacillus thiooxidans*, *Aspergillus* sp., *Pseudomonas* sp., *Mycococcus* sp., *Penicillium* sp., *Clavibacter michiganensis subsp*. *insidiosus*
*Lipopeptides and lipoproteins*	
Gramicidins	*Bacillus brevis*
Peptide lipids	*Bacillus licheniformis*
Serrawettin	*Serratia marcescens*
Surfactin, subtilysin, subsporin	*Bacillus subtilis*
Lichenysin G	*Bacillus licheniformis* IM1307
Amphomycin	*Streptomyces canus*
Globomycin	*Streptomyces globocacience*
Bacillomycin L	*Bacillus subtilis*
Iturin A	*Bacillus subtilis*
Putisolvin I and II	*Pseudomonas putida*
Arthrofactin	*Arthobacter* sp*.*
Fengycin	*Bacillus thuringiensis* CMB26
Mycobacillin	Bacillus subtilis
*Polymeric biosurfactants*	
Emulsan	*Acinetobacter caloaceticus* RAG-1,* Arethrobacter calcoaceticus*
Biodispersan	*Acinetobacter caloaceticus* A
Liposan	*Candida lipolytica*
Alasan	*Acinetobacter calcoaceticus*
Protein PA	*Pseudomonas aeruginosa*
*Particulate biosurfactants*	
Membrane vesicles	*Acinetobacter* sp. HO1-N, *Acinetobacter calcoaceticus*
Fimbriae, whole cell	*Acinetobacter calcoaceticus*, Cyanobacteria

The functional properties of biosurfactants are mainly determined by their structures, such as the location and size of their functional groups. Rosenberg and Ron [23] classified biosurfactants into two groups: (1) low-molecular-weight surface active agents (LMW) called biosurfactants, which efficiently lower surface and interfacial tension; and (2) high molecular-weight polymers (HMW) mainly referred to as bioemulsifiers which are more effective as emulsion-stabilizing agents. The first group includes: glycolipides, lipopeptides and phospholipids, whereas the second group includes polymeric and particulate biosurfactants such as emulsan and alasan [24,25]. Most such biosurfactants derived from the various microbial sources are either anionic or neutral. Their hydrophobic moiety is composed of long-chain fatty acids or fatty acid derivatives, whereas the hydrophilic portion can be a carbohydrate, amino acid, phosphate or cyclic peptide [26]. Their chemical nature and the concentrations produced depend on the type of microorganism and the growth conditions. Many microorganisms producing biosurfactants have been isolated from contaminated soils, effluents and waste water sources [27]. 

**Figure 3:**
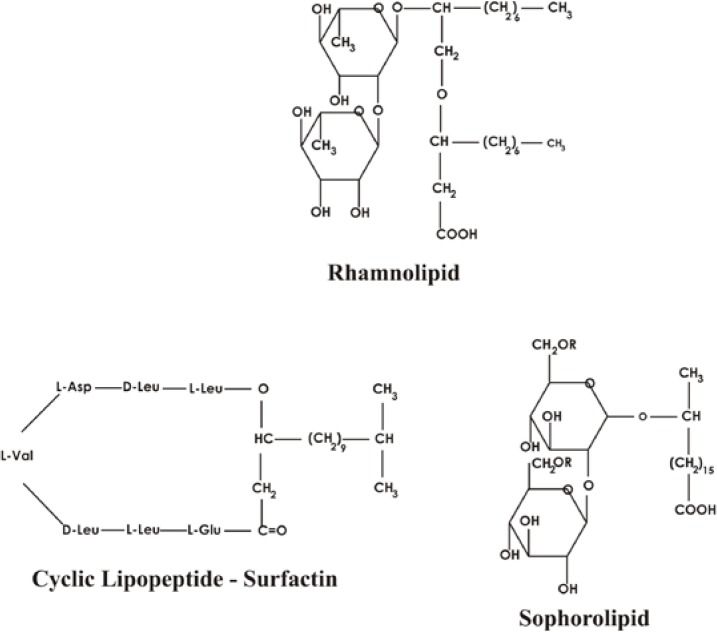
Major chemical types of biosurfactants produced by microorganisms.

Biosurfactants produced by microorganisms are also divided into the following classes: (1) hydroxylated and cross linked fatty acids (mycolic acids); (2) glycolipids—the best known among them are rhamnolipids, trehalolipids, mannosylerythritol lipids and sophorolipids; they are mainly carbohydrates linked to long-chain aliphatic acids or hydroxyaliphatic acids by an ester group; (3) lipopolysaccharides—that can have high molecular weights; (4) lipoproteins-lipopeptides—a large number of cyclic lipopeptides which are mainly produced by the *Bacillus* spp. and mainly are classified into four families: the surfactins, the iturins, the fengycins or plipastatins and the kurstakins; (5) fatty acids, phospholipids and neutral lipids—mainly produced by bacteria and yeast growing on n-alkanes, e.g. *Acinetobacter* sp. strain HO1-N and *R. erythropolis*; (6) polymeric biosurfactants—emulsan, liposan, alasan, lipomanan and other polysaccharide-protein complexes; *Acinetobacter calcoaceticus* RAG-1 produces an extracellular bioemulsifier; *Candida lipolytica* produces liposan—extracellular water-soluble emulsifier. The different chemical structures of biosurfactants are shown in Figure 3.

Biosurfactants are characterized by the following properties: critical micelle concentration (CMC), hydrophilic-lipophilic balance (HLB), charge and chemical structure [28]. At the critical micelle concentrations some physicochemical properties are changed and the value at this concentration is commonly used to measure the efficiency of any biosurfactant. Efficient biosurfactants have a low critical micelle concentration, which means that lower concentration of biosurfactant is required to decrease the surface tension. Micelle formation enables biosurfactants to reduce the surface and interfacial tensions, and increase the solubility and bioavailability of hydrophobic organic compounds [29]. The surface tension (ST) and interfacial tension (IT) are therefore important properties of biosurfactants. Molecules of water are held together by strong intermolecular forces build the high tension on the surface, which is 72 mN/m. When the biosurfactant is added to the water the surface tension is reduced. For example, surfactin produced by *Bacillus* spp., rhamnolipids produced by *Pseudomonas aeruginosa* and sophorolipids produced by *Starmerella bombicola* at the CMC reduces surface tension to approximately 30 mN/m. Similarly, the hexadecane-water interfacial tension is reduced from ≈40 to ≈1 mN/m. 

The hydrophilic-lipophilic balance (HLB) is another important parameter of biosurfactants characterizing their properties such as conductivity, viscosity, density, osmotic pressure, turbidity. This term was first described for nonionic surfactants [30]. The HLB value is a measure that indicates whether a biosurfactant is inclined to produce a water-in-oil or oil-in-water emulsion and is represented by a scale from 0 to 20, with lower values for products leading to water in oil emulsion formation and higher values for oil in water emulsion formation characteristics. This factor can be used to determine the most appropriate application for a particular biosurfactant. Emulsifiers with low HLB are lipophilic and stabilize water-in-oil emulsions, whereas emulsifiers with high HLB have the opposite effect and confer better water solubility. The HLB scale indicates the ability of biosurfactant to form emulsions of water-in-oil or oil-in-water. 

Biosurfactants offer many advantages over their chemical similitudes, including the following:

(1) Biodegradability—owing to the low toxicity and simple chemical structure, the compounds do not persist in the environment, are easily degraded and therefore do not accumulate in the environment [31];
(2) Biocompatibility and digestibility, which allows their unconstrained application in cosmetics, pharmaceuticals and as functional food additives [32];
(3) Availability of raw materials—biosurfactants can be produced from relatively cheap raw materials, which are available in large quantities [33];
(4) Acceptable production economics—most biosurfactants can be produced using sustainable substrates, and even using some industrial wastes and by-products, which represents a promising area for bulk production, which is important for use as detergents and cleaning products, or petroleum-related field technologies such as microbial enhanced oil recovery—MEOR [34];
(5) Use in environmental biotechnology—biodegradation and detoxification of industrial effluents, bioremdiation of contaminated soils, control of oil-spills, processes for stabilization of industrial emulsions [21];
(6) Specificity—due to specific functional groups in some biosurfactant molecules, they can used in detoxification of specific pollutants, de-emulsification of industrial emulsions, development of specific pharmaceutical and food applications [32];
(7) Effectiveness at extreme temperatures, pH, and salinity [35].

With such advantages and suitability for many industrial applications, many authors predict that biosurfactants will become increasingly attractive as multifunctional materials for future utilization by various industries [27].

## 4. Role of Biosurfactants in Biosynthesis of Metallic Nanoparticles

Recognizing the importance of developing eco-friendly methods for the synthesis of biologically active nanoparticles, scientists have recently started looking into research relating to the synthesis of metallic nanoparticles with the additional use of biosurfactants as capping agents [36,37,38,39]. It was observed that biosurfactants produced by microorganisms can play a very important role in aggregation and stabilization process. Biosurfactant use has therefore now emerged as a green alternative for enhancing both nanoparticles synthesis and stabilization. One of the modes of action is through adsorbing onto metallic nanoparticles (MeNPs), surface stabilizing the nanoparticles and preventing subsequent aggregation. The mechanism of surfactant adsorption depends on the type of surfactant (ionic, non-ionic, polymeric, *etc.*) and the thickness of the adsorbed layer [40,41,42]. So far no comparative study has been published concerning the influence of the biosurfactants’ nature and composition on the properties and ability to control metal NPs biosynthetic formation process. Different commercial biosurfactants as well as laboratory produced samples have been examined as stabilizer and modifier in the synthesis of metallic nanoparticles. 

### 4.1. Glycolipids Biosurfactants Produced Nanoparticles

Xie *et al.* [43] introduced the possibility of synthesizing silver nanoparticles in water-in-oil microemulsion stabilized by commercial rhamnolipid. The particles were initially synthesized using NaBH_4_ as reducing agent in rhamnolipid reverse micelles and were extracted from the micellar solution to disperse in heptane. The obtained silver nanoparticles were characterized by UV-vis absorption spectrum and were stabilized for at least two months. Transmission electron microscopy (TEM) and scanning electron microscope (SEM) showed that the silver nanoparticles obtained in the system were spherical and uniform. Palanisamy [44] also used commercial rhamnolipid as a stabilizer of NiO nanorods. The nanoparticles were synthesized using two different microemulsion approaches. In the first approach the microemulsion was prepared by dissolving commercial rhamnolipid in heptane and then adding NiCl_2_·H_2_O solution to the mixture with continuous stirring. In the second approach the microemulsion was prepared by adding NH_4_OH instead of nickel chloride solution. Microemulsions 1 and 2 were mixed together. The precipitated nickel hydroxide was separated by centrifugation and then washed with ethanol to remove biosurfactant and heptane. The nickel hydroxide precipitate was dried at 600 °C for 3 h to covert Ni(OH)_2_ to NiO. The produced nanorods were found to be approximately 22 nm in diameter and 150–250 nm in length. The morphology of the synthesized nanoparticles depended on the pH. 

A similar procedure was applied by Palanisamy and Raichur [45] to obtain spherical nickel oxide nanoparticles with uniform distribution. A method using rhamnolipid for conventional microemulsion production technique was reported. Increasing the pH of the solution decreased the size of nanoparticles. NiO nanoparticles of 86, 63 and 47 nm were obtained at pH values of 11, 12 and 12.5, respectively. Ganesh *et al.* [46] also reported developing a method to synthesize silver nanoparticles using purified rhamnolipids isolated from the culture supernatant of *Pseudomonas aeruginosa*. The silver nanoparticles synthesis was achieved with a purified rhamnolipid mixture used separately to form reverse micelles with aqueous silver nitrate (AgNO_3_) or aqueous sodium borohydride (NaBH_4_) solution. The first reaction was carried out by mixing aqueous silver nitrate, purified rhamnolipids and *n*-butanol/*n*-heptane until reverse micelles were produced. The second reaction consisted of the same quantities of solvents, rhamnolipid and aqueous borohyride. The two samples of reverse micelles were mixed and then silver nanoparticles were precipitated and separated by centrifugation. The nanoparticles obtained were mono-dispersed, had a spherical shape and an average particle size of 15 nm. The produced silver nanoparticles had significant antimicrobial activity against both Gram-Negative and Gram-Positive microbial pathogens. 

Narayanan *et al.* [47] also used water-soluble rhamnolipids produced by *Pseudomonas aeruginosa* for capping ZnS nanoparticles and highlighted the metal affinity, size and optical properties of the produced rhamnolipid-capped spherical ZnS nanoparticles. They were synthesized in an aqueous environment without any organic solvent and were both stable and water soluble. The FT-IR spectra obtained for rhamnolipid-capped ZnS nanoparticles showed an affinity between COOH of rhamnolipid and metal ions. The specific measurements revealed uniformity in size and a narrow size dispersion of the nanospheres with a radius range of about 4.5 nm.

Worakitsiri *et al.* [48] also used biosurfactants in organic nanoparticles synthesis. They used rhamnolipids as a soft, easily removable template in the synthesis of polyaniline (PANI) nano-fibers and nanotubes. Oxidative polymerization of aniline as a starting monomer was applied while using hydrochloric acid as a dopant agent and ammonium peroxodisulfate as an oxidant. In comparison to the conventionally synthesized polyaniline, the rhamnolipids associated polyaniline synthesis showed uniform morphology and size with higher crystallinity and electrical conductivity. They concluded that rhamnolipids were good candidates for the template synthesis of high conductive polymeric nanoparticles with controllable morphology and size. It was also interesting to note that the chemical surfactant-associated synthesis of polyaniline nanostructures using sodium dodecylsulphate (SDS) and nonylphenol ethoxylate (NP-9) had less promising product and results [48].

Rhamnolipids produced by *Pseudomonas aeruginosa* strain BS-161R was also used by Kumar and Mamidyala [49] in the extracellular synthesis of silver nanoparticles. The reduction of the silver ions was due to the enzyme nitrate reductase. They suggested that the nanoparticles were stabilized by rhamnolipid present in the culture supernatant. Another unidentified glycolipid produced by a sponge-associated marine *Brevibacterium casei* MSA19 was reported by Kiran *et al.* [38] as a suitable stabilizer in Ag nanoparticles biosynthesis under solid-state fermentation. Similar to other observations reported for rhamnolipids produced nanoparticles were stable for two months and the biosurfactant acted as stabilization agent and prevented the formation of aggregates. Saikia *et al.* [50] described a new role of rhamnolipid in the protection of silver nanoparticles against salt. Rhamnolipids produced by a *Pseudomonas aeruginosa* strain were used in testing the stability of colloidal silver nanoparticles (AgNPs) with respect to salt concentrations (2–60 mg NaCl/mL). It was suggested that rhamnolipid might be a potent stabilizer of the AgNPs in colloidal form. In absence of the rhamnolipid, the NaCl reacted with AgNPs to produce AgCl. 

Hazra *et al.* [51] also reported the biosynthesis of rhamnolipid biosurfactant capped zinc sulphide (ZnS) nanoparticles, its structural characterization, biocompatibility, cytotoxicity assessment and its applicability as a nanophotocatalyst for textile azo dye degradation were investigated. The purified rhamnolipid at 1.2-fold CMC was used directly as a capping and stabilizing agent in the reaction mixture during the synthesis of ZnS-NPs. They noted that capping the nanoparticles using biosurfactants reduced their toxicity and that the efficiency of ZnS nanoparticles in brown textile dye degradation/decolorization was quite high. The authors suggested that biosurfactant capped ZnS nanoparticles had a promising future for the generation nanophotocatalysts. 

Similar observations were also reported for sophorolipids use as cobalt nanoparticles and new water-soluble capping agents by Kasture *et al.* [52]. The oleic acid-derived sophorolipids were produced by growing yeast cell *Starmerella bombicola*. The produced cobalt nanoparticles showed good stability and super paramagnetic properties. The particles were reported to be polydisperse and well separated. The average particle size was around 50 nm. The authors’ main interest was in using the sophorolipid-capped Co nanoparticles to generate biocompatible particle surfaces by attachment of bioactive molecules such as lectins or glycosidases for medicinal and diagnostic applications.

Further reports by Kasture *et al.* [53] on using sophorolipids as a capping and reducing agent in silver nanoparticle synthesis carried out at room temperature, 40, 60, 80 and 100 °C were also reported. Two types of sophorolipids mixtures were produced either on oleic acid or linoleic acid which may affect the fatty acid chain length for the hydrophobic moiety of the sophorolipid biosurfactant molecule. At the lower temperatures, larger particles with broad size distributions were obtained. At increasing temperatures, the silver nanoparticles became smaller with a narrow size distribution. When linoleic acid produced sophorolipids were used in the biosynthesis larger ZnO particles were obtained in comparison to when oleic acid produced sophorolipids were used, which indicates a direct role for the biosurfactant molecules size and distributions in the nanoparticles formation.

Functional iron nanoparticles have also been synthesized using a sopholipids [54]. The acidic forms of sophorolipids were suitable compounds for use as surface complexing agents in the synthesis of iron oxide nanoparticles. Dynamic Light Scattering (DLS) experiments indicated that sophorolipids-derived nanoparticles had excellent colloidal stability both in water and in salt-containing aqueous solutions. The average sizes of the sophorolipids-functionalized iron oxide nanoparticles ranged between 10 and 30 nm in water and they did not become larger than 30 nm in water and KCl solutions (at 0.01 and 2 M). The size for most of the largest aggregates was generally below 100 nm, making sophorolipids interesting candidates for further studies on surface capping agents of nanoparticles for biomedical applications. Baccile *et al.* [54] suggest that the accessibility of the sophorose group at the surface of the nanoparticles is an important issue for biocompatibility properties. It was shown that sophorose layer coated the iron oxide nanoparticles. 

### 4.2. Lipopeptides Biosurfactants Produced Nanoparticles

Lipopeptides biosurfactants have also been reported used in nanoparticles synthesis. Reddy *et al.* [36,37] used surfactin as the template and stabilizing agent in the synthesis of silver and gold nanoparticles. The synthesis of stable gold nanoparticles by the reduction of aqueous AuCl_4_ using sodium borohydrate in the presence of surfactin produced by *Bacillus subtilis* was described [36]. Surfactin was recovered from the culture supernatant by foam fractionation and added to the pale yellow color chloroaurate solution which turned to red-purple, indicating the change in the metal oxidation state and the formation of gold nanoparticles. The nanoparticles were synthetized at the different pH values 5, 7 and 9 and at both 4 °C and at room temperature. They reported the synthesis of stable nanoparticles at pH 7 and 9 remaining intact for 2 months, while aggregation of nanoparticles synthesized at pH 5 occurred within 24 h. They also noted that mean particle size decreased at higher pH values. The nanoparticles synthesized at room temperature were monodispersed and were more uniform as compared with those produced at 4 °C. 

**Figure 4:**
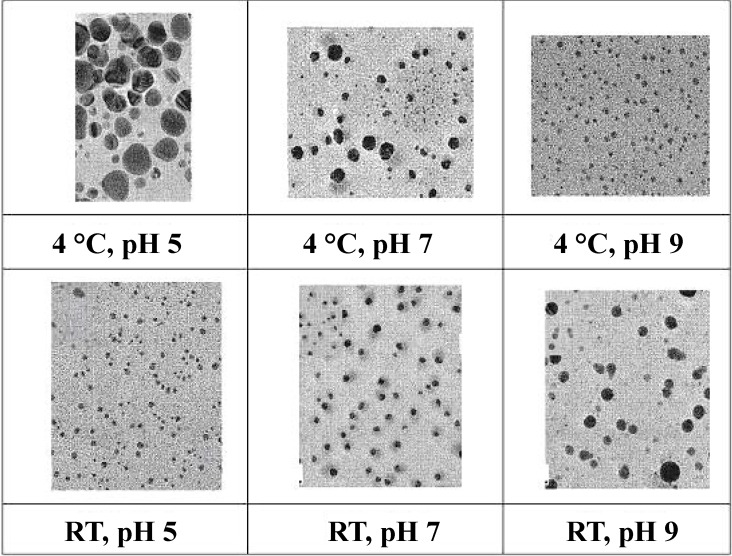
TEM (transmission electron microscopy) micrographs of silver nanoparticles synthetized at 4°C and room temperature (RT) at pH values 5, 7 and 9; modified from [37] with permission from Elsevier, copyright 2009.

In their second paper, Reddy and co-workers [37] used commercial surfactin to stabilize the formation of silver nanoparticles. A solution of AgNO_3_ was mixed with NaBH_4_ in the presence of surfactin. The produced nanoparticles were stable for 2 months. Their size also decreased with increase of pH from 5 to 9 at 4 °C (Figure 4). In contrast, at room temperature the size of the nanoparticles increased with increasing pH value (5 < 7 < 9). The shape of the silver nanoparticles was found to be more uniform as compared with their counterparts synthesized at 4 °C.

Surfactin used as a templating-agent stabilizer was also successfully employed to prepare nanostructured zinc oxide rose-like arrangements using the precipitation method [55]. The average thicknesses of the petals in the rose-like structures were between 9 and 13 nm. The morphology, density and thickness of the petals in the zinc oxide structures were significantly influenced by the concentration of surfactin used in the preparation. The thickness of the petal decreased with increasing of surfactin concentration. They also reported on the enhanced photocalalytic effects and properties of ZnO nanoparticles on the photodegradation of methylene blue under UV irradiation. Surfactin produced by *Bacillus amyloliquifaciens* was also used by Singh *et al.* [39] to synthesis stable cadmium sulfide nanoparticles (CdS-NPs). The yield of the surfactin extracted from the bacteria was determined to be 160 mg/L. This crude surfactin biosurfactant was directly used in synthesis of CdS nanoparticles and was reported as having a significant role in stabilizing and protecting the Cd-NPs. The produced nanoparticles covered with surfactin were stable for up to six months without any obvious change in their structure.

### 4.3. Chemical Surfactants and Nanoparticles

Kvitek *et al.* [41] investigated the influence of various chemically synthesized surfactants: anionic surfactants—SDS and Tween 80 and non-ionic surfactants from the Brij detergents and polymers: polyethyleneglycols (PEG) and polyvinylpyrrolidone (PVP) on aggregation stability and antibacterial activity of silver nanoparticles which were synthesized by the modified Tollens process using d-maltose as a reducing agent. Among the large scale of various surfactants and polymers, SDS, Tween 80 and PVP were found to act as the best stabilizers with an added advantages in the enhancement of the antibacterial activity of the modified Ag-NPs. They also attempted to describe the mechanism of surface interaction of surfactants and polymers with silver NPs and the reasons of the antibacterial activity. In the case of the Ag-NPs interaction with the ionic surfactants, the mechanism of the surfactant adsorption was not completely clarified. Earlier however, Chen and Yeh [42] suggested a possible mechanism of organization of SDS molecules on the NP surface in which the hydrophilic groups of the surfactant molecules are adsorbed on the silver NP surface and the hydrophobic tails are directed outward to form the first layer. Consequently, a counter-layer is oriented in the opposite way resulting in interpenetration of the surfactant hydrophobic tails between the two layers with hydrophilic groups headed outward. In the case of the silver NPs modified with SDS, they correlated the best surface stabilization with high antibacterial activity by the ability of SDS to increase the permeability of the cell wall or to disrupt the cell wall in Gram positive strains. The antibacterial and antiviral activity of silver, silver ions and silver nanoparticles is known and widely applied [56,57]. 

## 5. Antimicrobial and Cellular Activity of Nanoparticles

Both surfactin and other glycolipids biosurfactants have been reported to exhibit significant antimicrobial, antitumor activity, and anti-proliferative actions against many cancer cell lines [31,56]. There are several reports on the use and production of *Bacillus* bacteria and surfactin for controlling bacterial, mold, and fungal pathogens [58,59,60]. Recent studies have confirmed that specially formulated metal nanoparticles have a good antimicrobial activity and that nanoparticles-based antimicrobial formulations could be effective bactericidal and fungicidal materials [61,62,63,64]. The fundamental application problem of silver NPs is connected the efficient stability of their dispersions. This leads to the prevention of the aggregation process which causes a loss in their antibacterial activity.

The biological methods for producing nanoparticles are still at a developing stage. Many efforts should be made to improve the biosynthesis process and to understand the relations between physico-chemical and antimicrobial properties of metallic nanoparticles. The size, shape and controlled dispersion of Ag nanoparticles play a vital role in determining the physical, chemical and biological properties and their application in environmental, biotechnological and biomedical fields. Rapid and green synthetic methods using different microorganisms and biosurfactants have shown a great potential of Ag-NPs synthesis. A brief of nanoparticles synthesis using microorganisms such as bacteria, fungi, actinomycetes and yeast has been introduced in the literature. Most of the biosynthesis mechanisms have not been identified yet.

Better control of particle size and monodispersity of nanoparticles synthesis by microbes is still being sought. The influence of process parameters (concentration, temperature, pH) on Ag-NPs parameters (size, shape, distribution of sizes) and possibility of controlling biosynthesis by changing the above mentioned parameters should be investigated. The impact of microbes, growth media and synthesis conditions which are probably responsible for physico-chemical and biological properties of metallic nanoparticles are still not understood.

The biological activity is very important for further applications of metallic nanoparticles. The antimicrobial activities of nanoparticles in combination with biosurfactants are still unclear. There is growing demand for an environmentally benign synthesis process for metal nanoparticles, owing to the stringent regulations imposed by legislation on the use of toxic chemicals and emission of greenhouse gases. In response to the increasing demand for clean, nontoxic, and environmentally friendly methods for the synthesis of metallic nanoparticles, biosynthetic methods employing either microorganisms or plant extracts have been developed as environmentally sustainable alternatives to chemical and physical synthetic procedures.

## 6. Conclusions and Future Perspective

Biological systems; bacteria, fungi, actinomycetes, and algae have many opportunities for utilization in nanotechnology, especially in the development of a reliable and eco-friendly processes for the synthesis of metallic nanoparticles. The rich microbial diversity points to their innate potential for acting as potential bio-factories for nanoparticles synthesis. However, the biochemical and molecular mechanisms of biosynthesis of metallic nanoparticles need to be better understood to improve the rate of synthesis and monodispersity of the product. Characterization of the role biosurfactants play in the biosynthesis and their influence on the shape, size, dispersion and properties of metallic nanoparticles are required to elucidate the mechanisms that mediate microbial synthesis and to allow control of size, shape and crystallinity of the produced nanoparticles. Future research on the role of biosurfactants in biosynthesis of nanoparticles with unique properties is of great importance for specific applications in chemistry, medicine, agriculture and electronics related industries. 
